# Topical Formulations Based on Ursolic Acid-Loaded Nanoemulgel with Potential Application in *Psoriasis* Treatment

**DOI:** 10.3390/pharmaceutics15112559

**Published:** 2023-10-31

**Authors:** Małgorzata Miastkowska, Agnieszka Kulawik-Pióro, Elwira Lasoń, Karolina Śliwa, Magdalena Anna Malinowska, Elżbieta Sikora, Tomasz Kantyka, Ewa Bielecka, Anna Maksylewicz, Emilia Klimaszewska, Marta Ogorzałek, Małgorzata Tabaszewska, Łukasz Skoczylas, Krzysztof Nowak

**Affiliations:** 1 Department of Organic Chemistry and Technology, Faculty of Chemical Engineering and Technology, Cracow University of Technology, Warszawska 24, 31-155 Cracow, Poland; agnieszka.kulawik-pioro@pk.edu.pl (A.K.-P.); elwira.lason@pk.edu.pl (E.L.); karolina.sliwa@pk.edu.pl (K.Ś.); magdalena.malinowska@pk.edu.pl (M.A.M.); elzbieta.sikora@pk.edu.pl (E.S.); 2 Malopolska Centre of Biotechnology, Jagiellonian University, 30-387 Cracow, Poland; tomasz.kantyka@uj.edu.pl (T.K.); ewa.bielecka@uj.edu.pl (E.B.); anna.maksylewicz@uj.edu.pl (A.M.); 3 Department of Cosmetology, Faculty of Medical Sciences and Health Sciences, Casimir Pulaski University of Radom, Chrobrego 27, 26-600 Radom, Poland; e.klimaszewska@uthrad.pl (E.K.); m.ogorzalek@uthrad.pl (M.O.); 4 Department of Fruit, Vegetable and Mushroom Processing, University of Agriculture in Krakow, Balicka 122, 30-149 Cracow, Poland; malgorzata.tabaszewska@urk.edu.pl (M.T.); lukasz.skoczylas@urk.edu.pl (Ł.S.); 5 Wellnanopharm, Jerzego Samuela Bandtkego 19, 30-129 Cracow, Poland; nowak.krzysztof@autograf.pl

**Keywords:** ursolic acid, nanoemulgel, hybrid systems, *psoriasis*, release kinetic

## Abstract

*Psoriasis* is a chronic disorder that causes a rash with itchy, scaly patches. It affects nearly 2–5% of the worldwide population and has a negative effect on patient quality of life. A variety of therapeutic approaches, e.g., glucocorticoid topical therapy, have shown limited efficacy with systemic adverse reactions. Therefore, novel therapeutic agents and physicochemical formulations are in constant need and should be obtained and tested in terms of effectiveness and minimization of side effects. For that reason, the aim of our study was to design and obtain various hybrid systems, nanoemulgel–macroemulsion and nanoemulgel–oleogel (bigel), as vehicles for ursolic acid (UA) and to verify their potential as topical formulations used in psoriasis treatment. Obtained topical formulations were characterized by conducting morphological, rheological, texture, and stability analysis. To determine the safety and effectiveness of the prepared ursolic acid carriers, in vitro studies on human keratinocyte cell-like HaCaT cells were performed with cytotoxicity analysis for individual components and each formulation. Moreover, a kinetic study of ursolic acid release from the obtained systems was conducted. All of the studied UA-loaded systems were well tolerated by keratinocyte cells and had suitable pH values and stability over time. The obtained formulations exhibit an apparent viscosity, ensuring the appropriate time of contact with the skin, ease of spreading, soft consistency, and adherence to the skin, which was confirmed by texture tests. The release of ursolic acid from each of the formulations is followed by a slow, controlled release according to the Korsmeyer–Peppas and Higuchi models. The elaborated systems could be considered suitable vehicles to deliver triterpene to psoriatic skin.

## 1. Introduction

*Psoriasis* is a relatively common inflammatory skin disease that affects nearly 2–5% of the worldwide population. The clinical outcome of *psoriasis* is characterized by an acute inflammatory reaction in the first phase of the disease, with further progression to the chronic form. It is estimated that the incidence of *psoriasis* in Northern Europe and the Scandinavian population is between 1.5 and 3%, affecting both men and women equally. The occurrence of *psoriasis* in the Caucasian population is estimated at 60 cases per 100,000 per year [1].

*Psoriasis* is distinguished by three typical histological features: epithelial hyperplasia, dilation, prominent blood vessels, and accumulation of inflammatory cells in the dermis. Inflammatory processes taking place in the body are identified by the appearance of skin symptoms, i.e., scaly eruptions and joint/tendon symptoms. Skin squamous is formed as a result of keratinization of disease foci; they vary in size, tend to coalesce, and most often occur on the extensor skin of the limbs (mainly elbows and knees), the area of the scalp covered with hair, as well as on the skin of the hands and feet [2,3,4,5,6].

The detailed molecular mechanism of *psoriasis* development is not clear. The dominant theories point to impairment in the functioning of the acquired immune system based on excessive activation of dendritic cells, Th1/Th17 lymphocytes, and production of relative cytokines: IL-17a, IL-23, TNFα, and INFγ. The cornerstone of psoriasis development is the accumulation of neutrophils, correlated with the local production of proinflammatory cytokines (TNFα, IL-17, and IL-23) and other inflammatory mediators (leukotrienes and other arachidonic acid derivatives), coupled with Th1 cell influx and dendritic cell activation. Therefore, the limitation of NfkB activation, a fundamental regulatory transcription factor in inflammation, hinders the psoriatic inflammatory state, regardless of the underlying mechanism [7,8,9].

Topical therapy is a first-line treatment for patients with *psoriasis* and mainly includes salicylic acid ointments (5–30% salicylic acid), urea ointments (10–20% urea concentration), brine ointment (5–10%), salicylic acid oil (5–7%; for the scalp), salicylic acid sulfur ointment, dithranol creams, or ointments. Other formulations contain plant tars (from pine, beech, and juniper; concentrations 5–20%), corticosteroids (in creams, ointments, and solutions), vitamin D_3_ and its derivatives, and retinoids (tazarotene 0.05–1% as ointment or gel) [2,3,6,10,11,12,13,14,15].

Salicylic acid, vitamin D analogs, and retinoids, with different mechanisms of action, are usually combined with topical corticosteroids. While vitamin D has mainly antiproliferative (epidermal) effects, corticosteroids have mainly anti-inflammatory (dermal) effects. Vitamin D analogs and corticosteroids are the combined topical agents of choice in *psoriasis*, showing superior efficacy when compared with monotherapy [1,16].

However, a variety of therapeutic approaches have shown limited efficacy with frequent side effects. Glucocorticoid topical therapy is known to be associated with systemic adverse reactions. Additionally, applying topical corticosteroids in a large surface area or long-term use of topical corticosteroids may lead to a higher concentration of corticosteroids in the blood, leading to systemic side effects. Preparations with high potency are contraindicated due to the risk of skin atrophy, striae rubrae distensae, perturbed cicatrization, etc. [16,17,18].

Nevertheless, a significant number of patients are not satisfied with the current therapy, both with its limited effectiveness and formulation of the drug, leaving them with no other physician recommendations [19]. Moreover, patients complain about notable adverse reactions to current topical therapies. The most common side effects are skin irritation, dryness, peeling, erythema, and edema. Patients also report cloth staining, unpleasant odor, or high viscosity of the preparations. The low effectiveness of the currently available preparations is related to the limited potency of the therapeutic compound but also to the low penetration into the skin and modest adherence of patients to the recommendations. Therefore, novel therapeutic agents and physicochemical formulations are in constant need and should be obtained and tested in terms of effectiveness and minimization of side effects [6,19].

Ursolic acid (3β-hydroxyurs-12-en-28-oic acid, UA) is a pentacyclic triterpenoid that got its name from the Latin name of bearberry (*Arctostaphylos uva ursi*), from which it was initially isolated (bearberry contains up to 0.75% ursolic acid). This compound is usually found in plants together with its isomer oleanolic acid (3β-hydroxy-olea-12-ene-28-oic acid). Apart from bearberry, ursolic acid is also found in rosemary, sage, thyme, marjoram, lemon balm, hyssop, and lavender. In vitro studies have demonstrated the antioxidative, antiaging, anticancer, antimicrobial, and anti-inflammatory effects of ursolic acid. Ursolic acid’s anticancer activity is related to its potential for apoptosis induction, which leads to the death of overly proliferating cancer cells while also limiting inflammatory reactions. Similar hyperproliferation of keratinocyte cells is typically observed in psoriatic lesions. Moreover, the anti-inflammatory features of UA include inhibition of the COX-2 and NFkB signaling pathways, which are overactivated in *psoriasis* development. UA downregulates the expression of proinflammatory cytokines such as IL-17, the main cytokine responsible for modulating the pathological response of Th17 lymphocytes in *psoriasis*. In addition, studies on the effect of triterpenoids on human proteolytic enzymes demonstrated ursolic acid inhibition of human neutrophil elastase, a protease crucial for neutrophil migration and accumulation in *psoriasis*. Considering the abovementioned effects that may be caused by ursolic acid, this compound can be regarded as a potential active substance in *psoriasis* treatment [20,21,22,23,24].

Designing novel drug delivery systems that can accurately and safely deliver a drug to a target in a controlled manner with maximum therapeutic effect continues to be the main focus of pharmaceutical and medical research. In recent years, there has been significant development of nanomedicine and nanotechnology in the treatment of skin disorders, including *psoriasis*. In particular, colloidal nanocarriers (nanoemulsions, nanocapsules, lipid nanoparticles, nanoemulgels, etc.) have gained much attention due to their biocompatibility, biodegradability, low toxicity, and ability to encapsulate hydrophobic active substances that are difficult to formulate, thereby increasing their bioavailability. Prolonged and controlled drug release, characteristic of colloidal nanocarriers, reduces the required amount of active substances and, consequently, eliminates potential side effects [25,26,27,28,29].

The developed formulations should also be characterized by specific rheological and mechanical properties. For example, they belong to the group of non-Newtonian shear-thinning fluids that show thixotropy, which means that the product does not spread over the entire surface of the skin, has a soft consistency, adheres to the skin, and facilitates the transport of the medicinal substance into its deeper layer. Moreover, the cosmetic aspect of the product should also be considered, e.g., a pleasant color, smell, appearance, nongreasy feeling on the skin, and ease of removal. The group of topical formulations with the abovementioned features are special types of polymeric gels [6]. A representative of such carriers is a combination of nanoemulsions and hydrogels referred to as nanoemulgels (NEGs). Nanoemulgels have improved the topical efficacy of various poorly permeable hydrophobic therapeutics [30]. As a prolonged release system, they allow us to extend the duration of the substance concentration in the therapeutic range. The active substance is released slowly into the skin by the cross-linked structure [31]. Moreover, it provides adequate rheological and sensory properties of the preparation and facilitates its application to the skin. The application of polymeric gels prolongs the contact time of the preparation with the skin and increases the skin hydration level by forming a hydrophilic film on the skin surface and reducing transepidermal water loss. This is a very important aspect of skin regeneration [32].

According to the best of our knowledge, there is no information in the literature about the use of hybrid systems based on nanoemulgels, including bigels, in the treatment of *psoriasis* [6]. For that reason, the aim of our study was to design and obtain novel, various hybrid systems, nanoemulgel–macroemulsion and nanoemulgel–oleogel (bigel), as vehicles for ursolic acid and to verify their potential as topical formulations used in *psoriasis* treatment. To optimize the formulation composition, cytotoxicity analysis of individual components and final preparations was used as the main parameter to assess tolerability in the analysis of biological activity.

## 2. Materials and Methods

### 2.1. Materials

The formulations of nanoemulgel (NG), cream (C), serum (S), balm (B), and oleogel (O) and their combination (hybrid systems) were developed as carriers for ursolic acid (UA). The ingredients (International Nomenclature of Cosmetic Ingredients—INCI name) applied to obtain the UA-loaded systems and their suppliers are shown in Table 1.

### 2.2. Cytotoxicity Test of Formulation Components

#### 2.2.1. Cell Culture

HaCaT, a spontaneously immortalized keratinocyte cell line from the adult human skin of a 62-year-old male (CLS Cell Lines Service GmbH, Eppelheim, Germany) was cultured in Dulbecco’s modified Eagle’s medium (DMEM) (Thermo Fisher, Waltham, MA, USA) containing 10% fetal bovine serum (FBS) (Thermo Fisher), 100 U/mL penicillin (Thermo Fisher), and 100 µg/mL streptomycin (Thermo Fisher) at 37 °C/5% CO_2_. Prior to each experiment, the medium was changed to DMEM without supplementation.

#### 2.2.2. Cell Viability Assay

HaCaT cells were seeded on 96-well plates (50,000 cells/well) one day prior to the experiment. The next day, dilutions of ursolic acid, formulation components, or full formulations in DMEM were applied to the cells. After 24 h of incubation, the medium was removed, and the cells were washed with PBS and incubated with 200 µL/well of 0.5 mg/mL thiazolyl blue tetrazolium bromide (Sigma-Aldrich, St. Louis, MO, USA) in DMEM for up to 20 min at 37 °C/5% CO_2_. The medium was removed, and formazan crystals were dissolved in 120 µL of isopropanol (POCH, Poland) acidified with 5 mM hydrochloric acid (POCH, Gliwice, Poland). Ninety microliters of samples from each well were transferred to a new, transparent 96-well plate. The absorbance of the samples was measured using a Spectra Max Gemini EM (Molecular Devices, San Jose, CA, USA) at 570 nm. The results were calculated in comparison to the untreated control (DMEM only).

### 2.3. Preparation of Formulations

The composition of the formulations is protected by the patent application P.441627 [33].

#### 2.3.1. Preparation of the Nanoemulgel

The composition of the ursolic acid-loaded nanoemulgel (NG) is presented in Table 2. To obtain the NG, a multistage procedure was applied. First, the day before mixing other ingredients, the hydrogelator was dispersed in an appropriate amount of water to form a hydrogel matrix (Phase A). The active ingredient (ursolic acid) was dissolved in a mixture of oil emollients and emulsifier (Phase B) by heating phase B up to the temperature T = 60 °C. The water solution of a preservative (Phase C) was prepared and heated to 60 °C. Then, the oil phase (B) and the water phase (C) were combined by mixing with a mechanical stirrer for 10 min at a stirrer speed of v = 500 rpm. The process was carried out at 60 °C. The prepared preemulsion was then homogenized to obtain the nanoemulsion (NE) using a probe-type sonicator (UP200 Ht, Hielscher, Germany) with a sonication power of 40 W, an amplitude of 69%, and a sonication time of 105 s. In the next step, the nanoemulsion (Phase B + C) was mixed with the hydrogel matrix (Phase A) at a temperature of 50 °C and stirred at a speed of 500 rpm until a homogeneous nanoemulgel with the appropriate consistency was obtained, and the ambient temperature was reached.

#### 2.3.2. Preparation of the Macroemulsions (Cream, Serum, Body Balm)

The general composition of the macroemulsions is presented in Table 3. The components of the oil (A) and water (B) phases were weighed and heated to 65 °C to obtain clear mixtures. While maintaining the abovementioned temperature, both phases were stirred (RW20 digital IKA mechanical stirrer; Poland v = 300 rpm) for 15 min until a homogeneous consistency was obtained. Stirring was continued at v = 500 rpm until room temperature was reached.

#### 2.3.3. Preparation of the Oleogel

The general composition of the oleogel is presented in Table 4.

Vegetable oil, oleogelator 1, and ursolic acid were weighed and heated to 60 °C. To accelerate the dissolution process of ursolic acid, an ultrasonic bath was used for 15 min. The mixture was gently heated in a water bath while stirring with a mechanical stirrer (RW20 digital IKA mechanical stirrer; Poland v = 200 rpm, maintaining the temperature at approx. 60 °C). Subsequently, the preweighed oleogelator 2 was added in small portions, and the obtained mixture was stirred and heated until the oleogelator was dissolved. Then, the heater was turned off, and stirring was continued until the mass reached a temperature of 25 °C.

#### 2.3.4. Preparation of Hybrid Systems (Nanoemulgel–Macroemulsions; Nanoemulgel–Oleogel)

Hybrid systems were obtained by combining the base formulation (cream, serum, balm, and oleogel) with nanoemulgel (Table 2) and homogenized using a high-speed stirrer. First, a previously developed macroemulsion (Table 3) was obtained. Then, the nanoemulgel was combined with each of the formulations (serum, cream, and balm) in various weight ratios (from 10:90 to 90:10 wt.) at 40 °C and stirred (500 rpm) until room temperature was reached. In the case of the nanoemulgel–oleogel or bigel (BG) system, nanoemulgel was added in small portions to the previously obtained oleogel. The mass ratio of oleogel to nanoemulgel was variable (from 5:95 to 20:80 wt%). The systems were mixed using an IKA mechanical stirrer, Poland (200 rpm). Mixing was carried out at a temperature of 25 °C while gradually increasing the rotational speed of the stirrer. After adding all of the nanoemulgel to the oleogel, it was stirred at 400 rpm for 15 min. After this time, the preservative (0.8% wt.) was added to the system and mixed together. The general composition of the hybrid systems is presented in Table 5.

### 2.4. Physicochemical Analysis of Formulations

#### 2.4.1. DLS Analysis

The mean droplet diameter and polydispersity index of the nanoemulsion contained in the nanoemulgel were measured with the dynamic light scattering (DLS) method (Zetasizer Nano ZS, Malvern Instruments, Malvern, UK) at 25 °C. To avoid multiple scattering effects, the samples were diluted with deionized water 1:10 (*v*/*v*) to diminish the opalescence before the measurement. The obtained parameters were the hydrodynamic diameter expressed as the z-average value of the samples as well as the polydispersity index (PDI). The analysis was performed three times (n = 3) to determine the mean values of the droplet size and standard deviation with 10 submeasurements each. The particle size and PDI of the nanoemulsion were measured immediately after preparation.

#### 2.4.2. Microscopic Analysis

The size and morphology of selected formulations were observed using an optical microscope (Nikon Eclipse E400, Nikon Corp., Tokyo, Japan). A drop of each sample was placed on a microscope slide and covered by a cover slip. An image of the sample was acquired using digital image processing software (Nikon software version 4.30, Micro Video Instruments Inc., Avon, MA, USA). Formulations were characterized by both bright field and polarized light microscopy. The general appearance of the emulsions was recorded by taking images using a digital camera (PowerShot SD1300IS, Canon, New York, NY, USA).

#### 2.4.3. SEM Analysis

The morphology of the nanoemulgel was analyzed by a scanning electron microscope (Mira3-FEG-SEM, Tescan, Czech Republic)) with a pole emission (Schottky emitter) equipped with an X-ray energy dispersive spectrometer EDX (Oxford Instruments, Singapore) and a cooling table (Peltier) operating in the temperature range from as low as −30 °C. The microscope operates in high, low, and variable vacuum modes. For SEM investigations, the samples were prepared by rapid freezing in liquid nitrogen followed by freeze-drying for 48 h.

#### 2.4.4. TEM Analysis

Additionally, the morphology of the nanoemulgel was investigated using a JEOL JEM 2100 HT transmission electron microscope (JEOL Ltd., Tokyo, Japan). Samples were collected on 300 mesh grids made from copper and additionally covered with formvar film. On each grid, 5 µL of sample was applied. The excess was removed using filter paper and left to dry at ambient temperature. Transmission electron microscopy was used for observation with an accelerating voltage of 80 kV. Images were taken using a 4 k × 4 k camera (TVIPS) equipped with EMMENU software ver. 4.0.9.87 (TVIPS GmbH, Gauting, Germany).

#### 2.4.5. Rheology Analysis

To determine the rheological properties of the obtained formulations, a Brookfield R/S rotational rheometer (LaboPlus, Warszawa, Poland) was used with a cone/plate measuring system (cones C25-1 and C75-2) at room temperature (25 °C). Viscosity tests were conducted with a variable shear rate within the range of 1–500 s^−1^. Each individual result was calculated as the average of 3 measurements.

#### 2.4.6. pH Analysis

The pH value of the obtained formulations was determined with a multifunctional measuring tool (Seven Multi by Mettler Toledo, Warszawa, Poland), which was equipped with a pH-measuring glass electrode. All measurements were performed in triplicate.

#### 2.4.7. Stability Analysis

The preliminary assessment of emulsion and bigel stability was carried out using the centrifuge method. The samples (2 g) were placed in a centrifuge (EBA 20, Hettich GmbH & Co. KG, Tuttlingen, Germany) for 10 min at a rotation frequency of 3500 rpm. For stable samples, accelerated aging tests were also carried out by placing them in an incubator (40 °C) for 24 h and then in a refrigerator (3 °C) for another 24 h. The procedure was repeated 3 times. The stability of the formulation was also determined by macroscopic (visual) observations after a three-month storage period at 25 °C and, additionally, using the Turbiscan LabCooloer apparatus (Formulation Inc., Uni-Export Instruments, Warszawa, Poland), enabling the characterization of physicochemical phenomena of the dispersion, e.g., particle migration and particle size changes. The measuring head of the device (a light source with two synchronized transmission and backscatter sensors) scans the sample moving from the bottom to the top of the vial, collecting data every 40 μm over the entire height of the sample (up to 50 mm). The test results are presented in the graph generated by Turbiscan LAB software version 2.0.0.19. The test results were interpreted on the basis of the TSI (Turbiscan Stability Index).

#### 2.4.8. Texture Analysis

Consistency tests of the obtained formulations were carried out using a Brookfield CT3 texture analyzer (LaboPlus, Warszawa, Poland). A spherical probe made of nylon TA43, 14 g, was used (immersion depth of the probe 10 mm at a constant head speed of 0.5 mm/s). The obtained results were recorded by the TexturePro CT program. In the profile texture analysis (TPA), hardness was determined, which was measured by the mass necessary to press the probe to a depth of 10 mm (the maximum force recorded during 1 test cycle) and the adhesion force, which was a measure of the sample’s adherence to the probe (mass that had to be applied to the probe to pull it out).

### 2.5. Kinetic Study of Ursolic Acid Release from the Obtained Systems

The formulations tested contained 0.1% pure ursolic acid (*w*/*w*), and all were composed of safe and eco-friendly cosmetic ingredients. Four forms of obtained products were tested for the release of triterpene: macroemulsion, nanoemulgel, macroemulsion/nanoemulgel hybrid (50/50, *w*/*w*), and bigel.

The release profiles of ursolic acid under in vitro conditions were determined by the dialysis bag method using thermostatic chambers. The membrane applied (Spectra Pore) was made from cellulose and was characterized by a molecular weight cutoff from 6 to 8 kDa. The loaded amount of the formulations was 3.00 ± 0.05 g. The receptor buffer applied in the experiment (200 mL), PBS (pH = 7.4)/ethanol solution (60/40, *v*/*v*) ensured efficient solubility of the triterpene compound and was stirred at 200 rpm. The experiment was performed at 32 ± 0.5 °C for 8 h. The receptor solution samples were taken every 15 min during the first hour of incubation and then every 60 min for the next 8 h.

The concentration of the released ursolic acid in the acceptor solution was determined by HPLC using a Dionex Ultimate 3000 DAD chromatograph equipped with a UV detector. An XBridge column (250 mm × 4.6 mm; 3.5 μm) with a precolumn was applied for the separation of the formulation components. The mobile phase was isocratic and consisted of ACN/H_2_O (80/20, *v*/*v*), and the flow rate was 0.5 mL/min. The sample injection volume was 20 μL, and the analysis time was 25 min. Finally, the peaks were integrated at a wavelength of 210 nm to determine the total concentrations of ursolic acid in the acceptor solution.

#### Evaluation of the Release Kinetics

The kinetic analysis of UA release was determined by linear regression of the in vitro release curves using four different models: Equation (1) zero order (cumulative amount (%) of drug released over time, Equation (2)), first order (log of cumulative amount (%) of drug released over time, Equation (3)), Higuchi (cumulative amount (%) of drug released over the square root of time, Equation (4)), and Korsmeyer–Peppas (log cumulative amount (%) of drug released over log time, Equation (4)). The diffusional release exponent *n* in Equation (4) was calculated from the slope of the straight line mathematical model best expressing the kinetic release profile was selected based on the highest value of the determination coefficient (R^2^) [34,35,36].

*Q*_*t*_ = *Q*_0_ + *K*_0_·*t* (1)

*logQ*_*t*_ = *logQ*_0_ + *K*_1_·*t* (2)

*Q*_*t*_ = Q_0_ + *K*_*H*_·*t*^1/2^ (3)

*Q*_*t*_ = *Q*_0_ + *K*_*HP*_·*t*^*n*^ (4)

where:

*Q*_*t*_—the amount of drug released in time t;
*Q*_0_—the initial amount of drug;
*K*_0_—zero-order kinetic constant;
*K*_1_—first-order kinetic constant;
*K_H_*—Higuchi kinetic constant;
*K_KP_*—Korsmeyer–Peppas release constant;
*n*—diffusional release exponent;
*t*—time.

### 2.6. Statistical Analysis

The results were analyzed with GraphPad Prism (GraphPad Prism, ver. 10.0.2, GraphPad Software LLC., Boston, MA, USA) software and are presented as the mean of each experiment ± SD. Statistical significance was evaluated with built-in one-way ANOVA. The results were considered statistically significant if *p* values ≤ 0.05. *—*p* = 0.05–0.011; **—*p* ≤ 0.01; ***—*p* ≤ 0.001; ****—*p* ≤ 0.0001.

## 3. Results

### 3.1. Screening of Formulation Components

To determine the safety and effectiveness of the prepared ursolic acid carriers, in vitro studies on human keratinocyte cell-like HaCaT cells were performed with cytotoxicity analysis. The individual components of the final formulations—hydrogel matrices, emulsifiers, and preservatives—were studied using the MTT assay.

**Figure 1:**
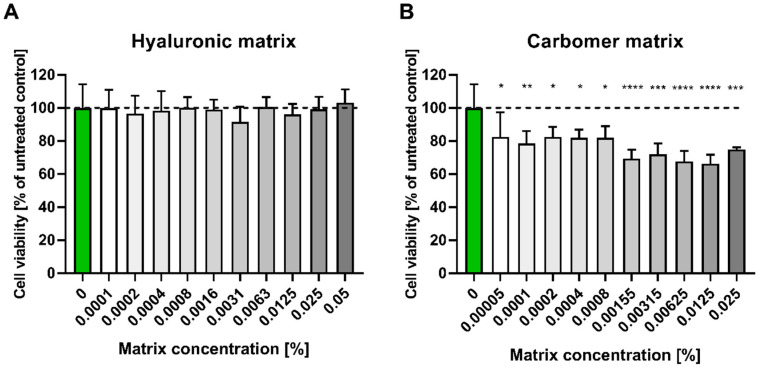
HaCaT cell viability test (MTT) in the presence of (**A**) hyaluronic and (**B**) carbomer matrices. HaCaT cells were incubated for 24 h with serial dilutions (0–0.05%) of hyaluronic and carbomer matrix prepared in DMEM. The results were calculated as the percentage of control cells incubated in the presence of DMEM alone and are shown as the mean ± SD. *—*p* = 0.05–0.011; **—*p* ≤ 0.01; ***—*p* ≤ 0.001; ****—*p* ≤ 0.0001. Green bar represents the unstimulated control; bar shading indicates the concentration gradient of the stimulant from the lowest (white) to the highest (dark).

The obtained results (Figure 1) indicated that the carbomer matrix showed higher cytotoxicity than the hyaluronic matrix. While the hyaluronic matrix was well tolerated up to a concentration of 0.05%, showing no statistically significant cytotoxicity, a 0.00005% concentration of the carbomer matrix led to an approximately 20% reduction in the viability of the tested cells.

**Figure 2:**
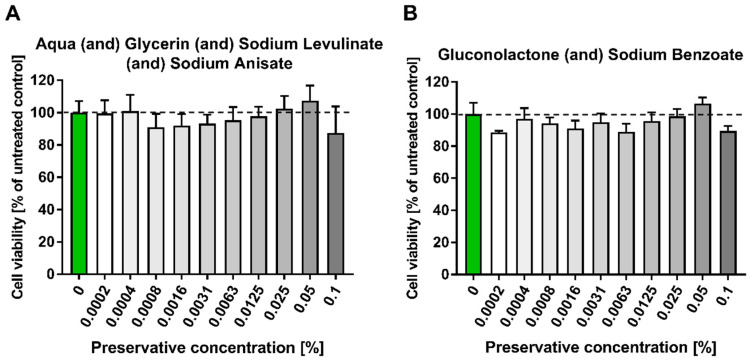
HaCaT cell viability test (MTT) in the presence of preservatives (**A**) aqua (and)/glycerin (and) sodium levulinate (and) sodium anisate; (**B**) gluconolactone (and) sodium benzoate. HaCaT cells were incubated for 24 h with serial dilutions (0–0.1%) of two commercial preservatives prepared in DMEM. The results were calculated as the percentage of control cells incubated in the presence of DMEM alone and are shown as the mean ± SD. Green bar represents the unstimulated control; bar shading indicates the concentration gradient of the stimulant from the lowest (white) to the highest (dark).

Both tested preservatives, aqua (and) glycerin (and) sodium levulinate (and) sodium anisate nor gluconolactone (and) sodium benzoate, had no negative effect on HaCaT cell viability in the concentration range of 0–0.1% (Figure 2).

The emulsifiers of natural origin were analyzed at a concentration range of 0–0.4%.

**Figure 3:**
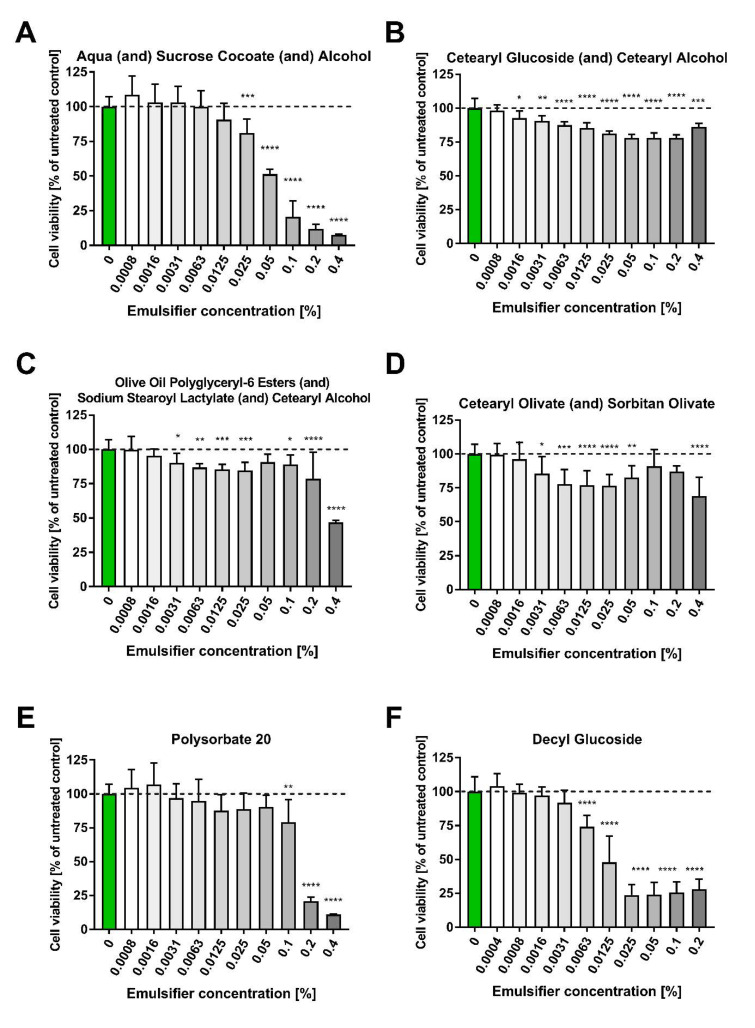
HaCaT cell viability test (MTT) in the presence of six types of emulsifiers: (**A**) aqua (and) sucrose cocoate (and) alcohol, (**B**) cetearyl glucoside (and) cetearyl alcohol, (**C**) olive oil polyglyceryl-6 esters (and) sodium stearoyl lactylate (and) cetearyl alcohol, (**D**) cetearyl olivate (and) sorbitan olivate, (**E**) polysorbate 20, and (**F**) decyl glucoside. HaCaT cells were incubated for 24 h with serial dilutions (0–0.4%) of six types of emulsifiers resuspended in DMEM. The results were calculated as the percentage of control cells incubated in the presence of DMEM alone and are shown as the mean ± SD. *—*p* = 0.05–0.011; **—*p* ≤ 0.01; ***—*p* ≤ 0.001; ****—*p* ≤ 0.0001. Green bar represents the unstimulated control; bar shading indicates the concentration gradient of the stimulant from the lowest (white) to the highest (dark).

The MTT analysis showed that the emulsifiers with the lowest cytotoxicity for HaCaT cells in the broadest concentration range were cetearyl glucoside (and) cetearyl alcohol (Figure 3B), olive oil polyglyceryl-6 esters (and) sodium stearoyl lactylate (and) cetearyl alcohol (Figure 3C) and cetearyl olivate (and) sorbitan olivate (Figure 3D).

### 3.2. Composition and Characterization of Formulations

#### 3.2.1. Morphology of Formulations

**Figure 4:**
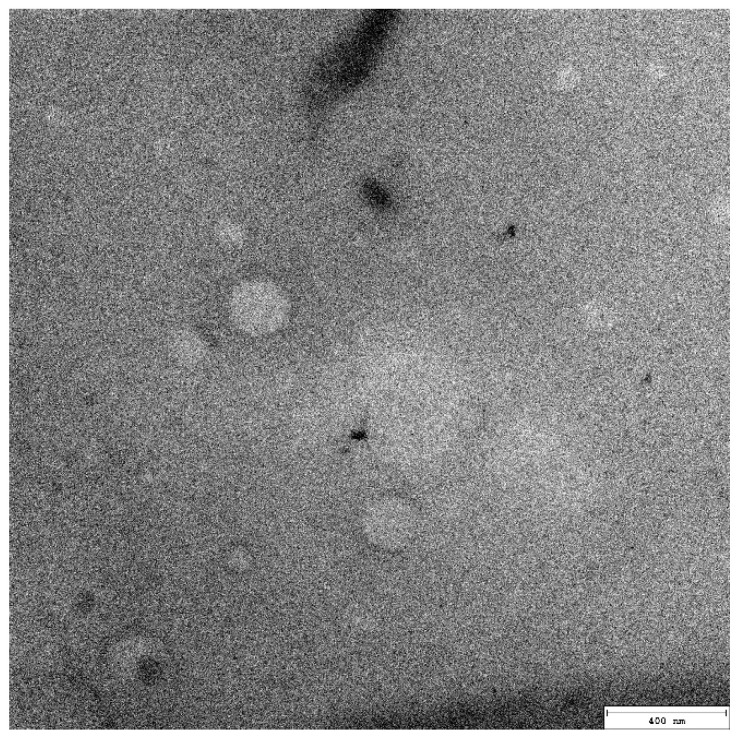
Transmission electron micrograph of nanoemulgel (NG).

The morphological TEM analysis (Figure 4) of the nanoemulgel revealed nanosized, spherical, and nonaggregated oil globules [37,38].

**Figure 5:**
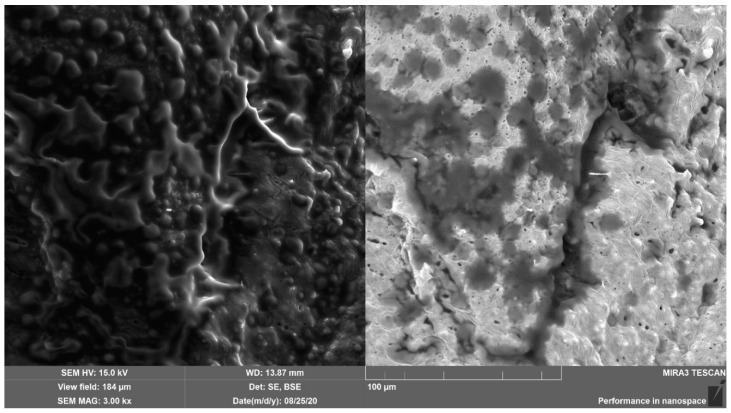
SEM images of the nanoemulgel (NG).

**Figure 6:**
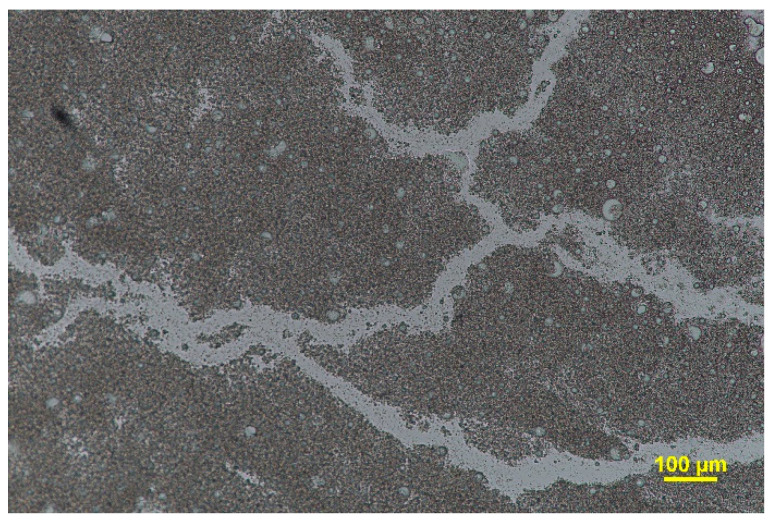
Optical micrograph of the nanoemulgel (NG).

The scanning electron microscopy (SEM) images (Figure 5) of the nanoemulgel, as well as optical micrographs (Figure 6), show a smooth and homogeneous surface of the nanoemulgel with fine oil droplets. The gel was stable without UA precipitation. Interconnected porous structures may provide sufficient space for high drug loading and movement of the drug throughout and enhance the drug release rate [39,40].

**Figure 7:**
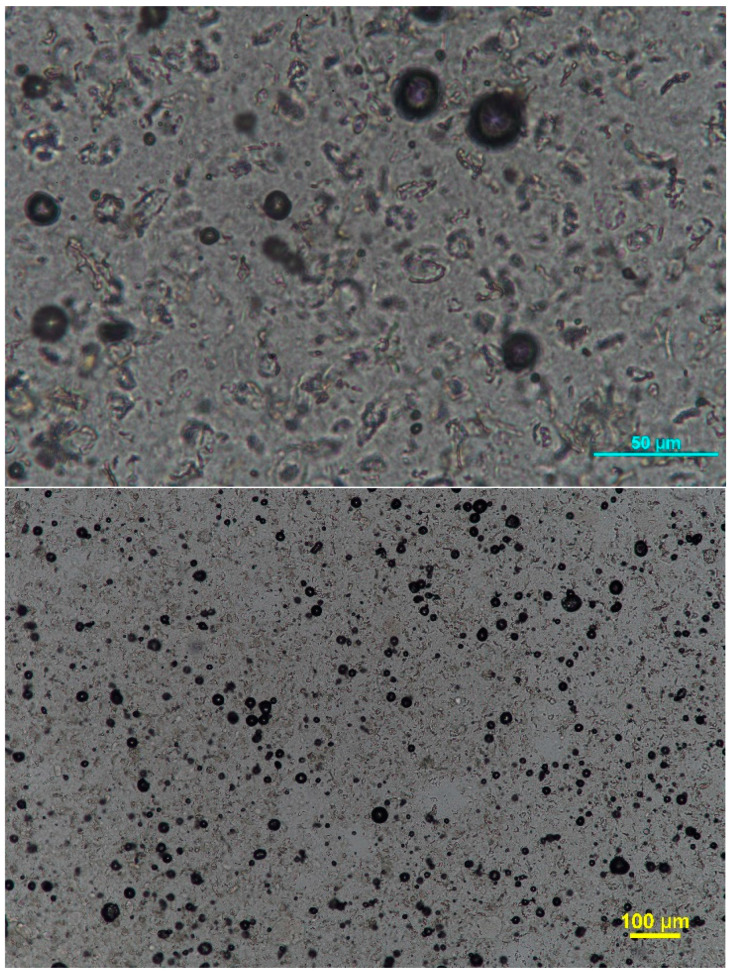
Optical micrographs of bigel (BG-1).

The analysis of the bigel micrographs (Figure 7) suggests the formation of a nanoemulgel-in-oleogel type of bigel. The dispersed spheres of the nanoemulgel have two-layered structure of hyaluronic acid molecules adsorbed on the surface of the nanoemulsion oil droplets in the oleogel [41,42]. According to the lower amount of nanoemulgel (5% *w*/*w*), two types of dispersed phases are observed: fibrous structures, which can be attributed to the network formed by the oleogelator, and irregularly shaped black globular bodies assigned to the presence of hydrophilic hyaluronic acid gel [43].

**Figure 8:**
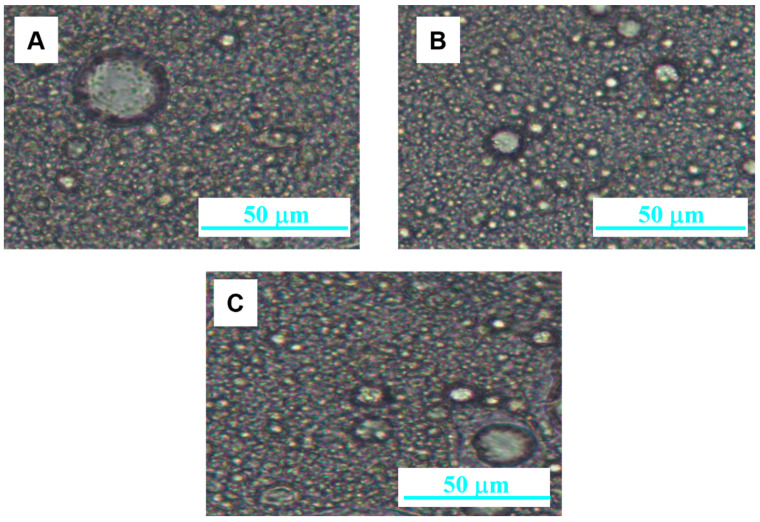
Optical micrographs of selected formulations: (**A**) sample NG:S-5; (**B**) sample NG:C-7; (**C**) sample NG:B-4.

Morphological analysis of a macroemulsion/nanoemulgel (Figure 8) combination confirmed highly dispersed systems of the nanoemulgel-thickened macroemulsion system.

#### 3.2.2. Physicochemical Properties of the Obtained Formulations

Based on the results of the physicochemical tests (pH, viscosity, particle size, stability) for all obtained formulations and their combinations with nanoemulgel, stable systems were selected for further research, for which no significant changes in the average particle size of the internal phase, viscosity, and pH were observed (Table 6).

The data in Table 6 show that the pH of the tested formulations after their production (t = 0) ranged from 4.05 to 6.05. The lowest pH was found in bigel (4.69 ± 0.1) because the pH of the oleogel was 4.05 ± 0.0, and the highest was found in balsam (6.05 ± 0.1). Despite the variable weight ratios of nanoemulgel:macroemulsion, the pH values for hybrid systems, combinations of nanoemulgel with serum, cream, and balm after production and after 3 months of storage are similar, and only a slight increase in these values was observed over time.

The lowest viscosity among all formulations was shown by nanoemulgel (1.20 ± 0.01 Pa∙s) and the highest by bigel (a system of two gels) (3.28 ± 0.17 Pa∙s). The addition of nanoemulgel to the macroemulsion slightly reduces the viscosity of the formulation. With an increase in the mass ratio of nanoemulgel:macroemulsion in favor of nanoemulgel, this decrease is more visible. For all formulations, a slight increase in viscosity is observed during storage. In the case of macroemulsions and their hybrid combinations, systems with lower viscosity were also characterized by a smaller average particle size. However, the addition of nanoemulgel did not significantly affect the average particle size of the internal phase of the macroemulsion (cream, serum, and balm), which was confirmed by microscopic analysis (Figure 8). The stabilization processes of the systems during storage slightly affected the average particle size after 3 months.

The stability of the formulation parameters, such as pH, average particle size, and viscosity, indicate that the obtained hybrid compounds are characterized by desirable features for topical formulations. Based on the obtained results, NG, S, C, and B preparations were selected for further research in addition to the following hybrid combinations: NG:S-5, NG:C-7, NG:B-4, and BG-1. The concentration of ursolic acid in each system was the same.

**Figure 9:**
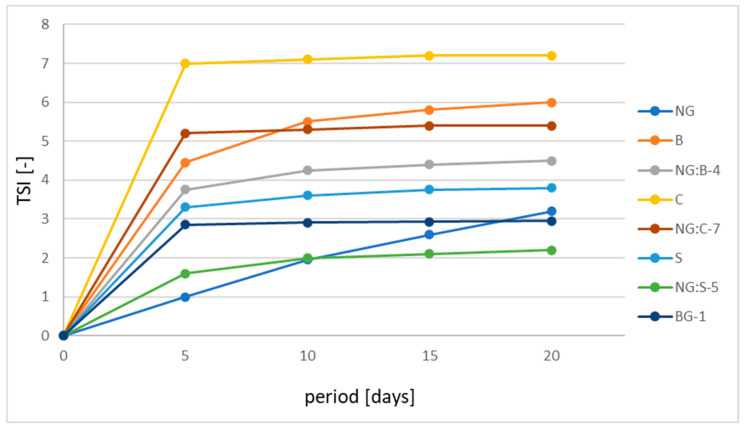
TSI value for all tested samples.

In the next stage of research, advanced stability tests were performed for the selected systems using the optical method, enabling the characterization of physicochemical properties of dispersion (particle migration, particle size change, flocculation, coalescence, sedimentation, creaming, and aggregation) detected at a very early stage without spoiling the structure of the system during the measurement. The stability of all tested formulations was compared using the obtained TSI index values. This dimensionless parameter defines the global stability of the product [44,45,46]. The calculation of the TSI is carried out on the basis of the transmitted light signal T and the back-reflected signal BS measurements. Then, all signal changes in the sample are summed to calculate the destabilization of a sample [47]. For this reason, this parameter is also called the instability factor in the literature [44,47,48]. The TSI values range from 1 to 100. It is assumed that the higher the TSI value is, the less stable the product [48,49,50]. Figure 9 shows the TSI values for the tested formulations.

Based on the obtained results (Figure 9), after 5 days of storing the samples at 40 °C, the lowest TSI value (1.0) was found for the nanoemulgel (NG), and the highest (7.0) was found for the cream (C). After 20 days of sample storage, the lowest TSI value of approximately 2.2 was found in the NG:S-5 formulation, a hybrid combination of serum and nanoemulgel, which had the best stability among the tested products. TSI values of approximately 3.0 were shown by the serum (S), nanoemulgel (NG), and bigel (BG-1). On the other hand, the highest TSI values were found in the case of the cream (C), hybrid combination cream–nanoemulgel (NG:C-7), balm (B), and combination of balm with nanoemulgel (NG:B-4). The TSI values for these products ranged from 4.5 to 7.0.

#### 3.2.3. Texture Profile

The physical nature of the formulations affects the user’s perception of the product. The texture analysis (including hardness and adhesion strength) of the selected formulations allows objective comparison of specific features of the formulation, which are usually determined by the user’s senses (usually subjectively) and significantly affect the willingness of people with skin problems to use them regularly and systematically.

**Figure 10:**
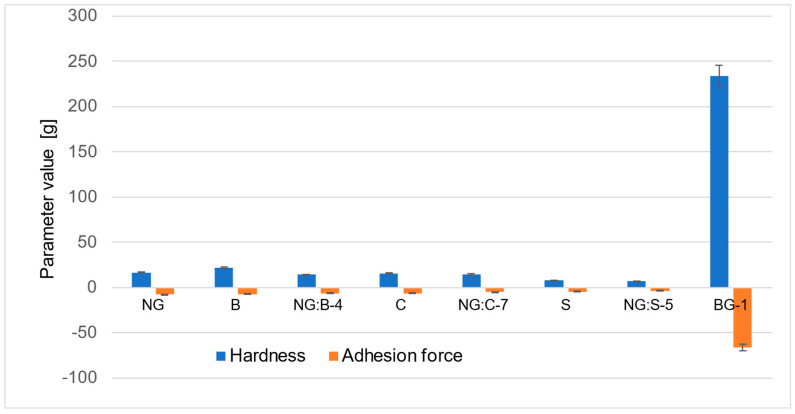
The average results of hardness and adhesion strength for the tested formulations.

Analyzing the results of the texture tests (Figure 10), it was observed that the bigel (a system of two gels) had the highest value of hardness (233.8 g). However, the hardness values for the other formulations ranged from 6.7 g to 21.5 g. According to the obtained results, a significant effect of the addition of nanoemulgel (NG) on the hardness of the developed macroemulsions was found. The addition of nanoemulgel (NG) to the emulsion system leads to a decrease in its hardness. For hybrid combinations of nanoemulgel with balm (NG:B-4), cream (NG:C-7), and serum (NG:S-5), lower hardness values were obtained compared to their identical formulations without nanoemulgel by 34%, 8.2%, and 14.1%, respectively.

The results of the adhesion force for the analyzed macroemulsions ranged from −3.5 g to −7.5 g. The serum with the addition of nanoemulgel (NG:S-5) had the highest adhesion force (−3.5 g) among all formulations, while the bigel had the lowest (−66.2 g). In the case of the results of the adhesion strength tests for hybrid combinations of nanoemulgel with serum, cream, and balm, a similar trend of changes was observed when compared to the results obtained from the hardness and viscosity tests. After adding nanoemulgel to the macroemulsion, the adhesion strength increased for the hybrid combinations NG:B-4, NG:C-7, and NG:S-5.

#### 3.2.4. Cytotoxicity Test of Formulations

**Figure 11:**
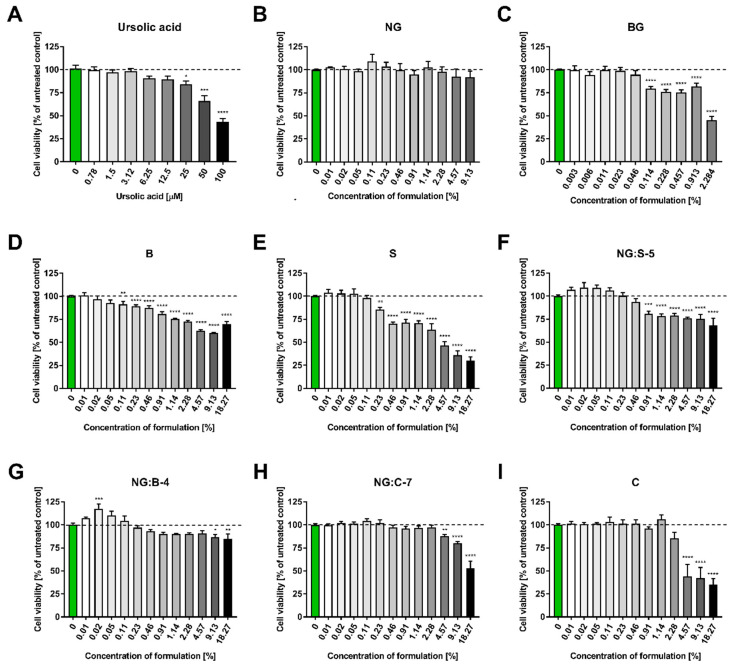
HaCaT cell viability test (MTT) in the presence of active substance alone—ursolic acid and eight formulations containing UA. HaCaT cells were incubated for 24 h with serial dilutions of (**A**) ursolic acid (0–50 µM) and eight types of cosmetic formulations (**B**) NG, (**C**) BG, (**D**) B, (**E**) S, (**F**) NG:S-5, (**G**) NG:B-4, (**H**) NG:C-7, and (**I**) C resuspended in DMEM. The exact concentrations for each formulation are marked on the graphs. Cell viability was assessed with the MTT assay. The results were calculated as the percentage of control cells incubated in the presence of DMEM alone and are shown as the mean ± SEM. *—*p* = 0.05–0.011; **—*p* ≤ 0.01; ***—*p* ≤ 0.001; ****—*p* ≤ 0.0001. Green bar represents the unstimulated control; bar shading indicates the concentration gradient of the stimulant from the lowest (white) to the highest (dark).

Full cosmetic formulations containing ursolic acid were prepared, tested on the HaCaT cell line to determine potential cytotoxicity, and compared to the active substance alone (UA). Cells at high confluence (>70%) were incubated for 24 h with serial dilutions of ursolic acid alone or with each formulation prepared in DMEM. In the case of the bigel (BG), which showed poor direct solubility in DMEM, the formulation was initially resuspended in DMSO and further diluted in a cell medium. The MTT assay showed no negative effect of ursolic acid on keratinocyte cell viability up to a concentration of 12.5 µM. Concentrations above 25 µM caused a decrease in cell viability by ~25% (IC25%), and the determined IC50% was above 50 µM. The majority of formulations were very well tolerated by the cells, as determined by IC25% values that were higher than those for ursolic acid alone (Table 7). The formulations NG:C-7, NG:B-4, NG, and C were characterized by the lowest cytotoxicity, as even a 10% concentration of formulation (containing 200 µM ursolic acid) did not decrease cell viability by more than 25% (Figure 11). Bigel (BG) and serum (S) samples showed higher cytotoxicity when compared to other formulations and even ursolic acid alone, as concentrations over 1 and 5% of each formulation reduced cell viability by 25%.

#### 3.2.5. Ursolic Acid Release Studies

**Figure 12:**
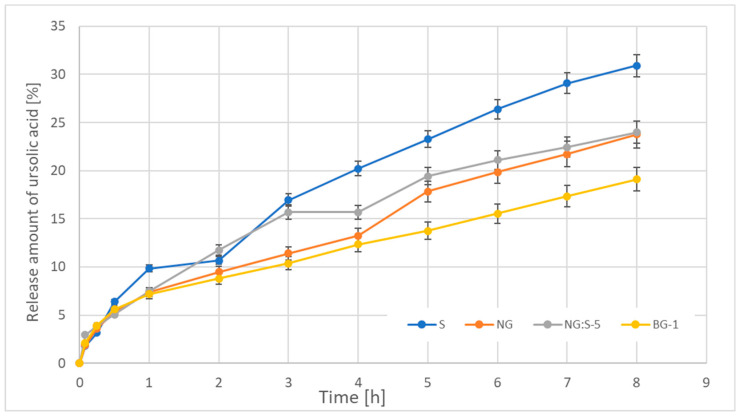
Release profiles of ursolic acid from serum (S) (as the representative sample of macroemulsions), nanoemulgel (NG), hybrid system of nanoemulgel and serum (NG:S-5), and bigel (BG-1). Values are expressed as the mean values (*n* = 3).

Some formulations, characterized by the highest stability due to the lack of particle size change over time (Table 6) and the lowest TSI (Figure 9), were selected for UA release tests. The in vitro release profiles of ursolic acid from the macroemulsion (serum was chosen as the representative sample of prepared macroemulsions), nanoemulgel (NG), hybrid system of nanoemulgel and serum (NG:S), and bigel (BG) are shown in Figure 12.

Based on the obtained results, it was found that the form of formulation used as the UA carrier influenced the active release profile. Within 8 h, approximately 31% of the active compound was released from the O/W macroemulsion (S), approximately 24% from the nanoemulgel (NG) and the hybrid system (NG:S-5), and approximately 19% from bigel (BG-1).

##### Kinetic Analysis of Ursolic Acid Release

According to the results obtained from the in vitro studies, the release of ursolic acid from the formulations was kinetically evaluated. Zero-order, first-order, Higuchi, and Korsmeyer–Peppas models were used for this purpose. The kinetic model parameters, including the determination coefficient corresponding to each mathematical model, are shown in Table 8.

As shown, the formulations did not follow zero-order or first-order kinetics. The release profiles could be best explained by the Higuchi and Korsmeyer–Peppas models.

## 4. Discussion

### 4.1. Composition and Characterization of Formulations

#### 4.1.1. Physicochemical Properties of Formulations

##### The pH of the Formulations

The pH values of the skin with dermatoses may differ from the typical pH range of 5–6 for healthy skin [51]. However, the pH of protective/healing formulations should be as close as possible to these “healthy” values to avoid skin irritation and impairment of the lipid barrier and maintain the integrity of the stratum corneum [51,52,53]. The low pH of the formulations applied to damaged skin also reduces the skin’s susceptibility to microorganisms and, thus, the rate of regeneration of damaged tissue [32]. All developed formulations were characterized by a pH appropriate for *psoriasis* skin.

##### Average Particle Size and Viscosity of the Formulation

Since the same type and concentration of emulsifier (4 wt%) was used to stabilize the macroemulsions, the observed differences in the average particle size and viscosity of the prepared hybrid formulations are related to the amount and type of oil phase used and to the content of nanoemulgel. The presence of liquid lipids in macroemulsions reduces their viscosity and causes smaller average particle sizes in the internal phase. Hybrid systems based on serum macroemulsion with the highest amount of liquid lipids (20% wt.) are less viscous, while systems containing semisolid lipids and/or solid lipids, i.e., the cream and balm, as well as their hybrid combinations, are more viscous and characterized by different particle sizes.

The addition of nanoemulgel into macroemulsion systems had no significant influence on droplet size but slightly decreased the viscosity of the formulation with an increasing nanoemulgel:macroemulsion mass ratio in favor of the nanoemulgel (Table 6). Nanoemulgel could lead to the formation of the gel network in the continuous phase, and the macroemulsion droplets might be located in the gel network [54].

The obtained formulations, thanks to the combination of physicochemical properties of both forms, such as macroemulsion and nanoemulgel, were characterized by apparent viscosity, ensuring the appropriate time of contact with the skin, ease of spreading, pleasant sensory sensations, and quick absorption. Such properties may encourage people suffering from *psoriasis* to regular and frequent use and, consequently, increase the therapeutic effect [55]. On the one hand, combining nanoemulgel with macroemulsions containing a high proportion of liquid lipids, semisolids, and solid lipids in their formula leads to obtaining topical formulations characterized by soothing, moisturizing, and nourishing properties. The soft consistency of the products also improves patients’ comfort during their application onto the skin and their effectiveness [55,56,57,58,59]. Both the polarity and physical state of emollients may affect the mechanisms of interaction of the products with the skin surface. Additionally, it influences the structural organization and organoleptic characteristics of the product [60]. On the other hand, the addition of the nanoemulgel, which belongs to the group of polymeric gels, may lead to prolonged contact time of preparations with the skin and increase its hydration level by forming a hydrophilic film on the skin surface [32].

##### Stability of Formulations

The results of the stability tests over time, including formulation parameters such as pH, average particle size, or viscosity (Table 6), indicated that all obtained systems for the treatment of *psoriasis* are characterized by stability over time. No significant changes in the parameters were observed during storage. The studies conducted by the optical method (static multiple light scattering) confirmed the product’s stability. Our results correlate well with those of other researchers. According to Ren et al., 2018 [45] and Manca et al., 2016 [61], the best stability is characterized by samples for which the ΔTSI values are lower than 0.4. This condition is met by the following samples: bigel (BG-1), serum (S), and cream (C). However, it should be noted that the cream had the highest initial TSI value (7.0). Nevertheless, such values also confirm the stability of the samples over time because, according to Śliwkowska [62], formulations with a TSI >10 are considered unstable. In addition, the TSI results obtained for the considered systems in this work are within the range of those obtained by Huang et al., 2021 concerning two cedarwood essential oil emulsions [63]. Similarly, the TSI value (approximately 2.3 to 5.3) in this study that confirmed product stability was also presented in the work by Nizioł-Łukaszewska et al., 2018 for body lotions [64]. In the case of results obtained by Kowalska et al., 2021, who studied emulsions with newly synthesized lipids stabilized with xanthan gum, TSI ranged from 1.35 to 8 [49].

#### 4.1.2. Texture Profile

The sensory properties and evaluation of the product’s texture play an integral role in the consumer’s perception of the formulation as a final product. Typical sensory properties of formulations applied topically to the skin are fragrance, color, uniformity, spreadability, or stickiness. Many sensory attributes correspond to the textural properties of the skin care product [65,66]. Texture analysis is increasingly used to characterize products in the food, cosmetic, and pharmaceutical industries [67]. The texture of the formulations was analyzed using two parameters: hardness and adhesion strength. The hardness is the maximum force recorded during the analysis, while the adhesive force is the lowest (negative) value recorded during the test [67,68].

In the case of the analyzed systems, adhesion is particularly important, as it is helpful in predicting the local residence time. Additionally, samples with higher hardness are more difficult to remove from the container or spread on the skin [53,69]. Moldovan et al. [70] conducted textural studies of emulsions stabilized by two different systems of emulsifiers. Formulation 1 (F1) was prepared using glycerol stearate and Ceteareth-25 as emulsifiers, while Formulation 2 (F2) was prepared using glyceryl stearate and potassium cetyl phosphate, all other ingredients remaining the same. They showed that the addition of a mixture of glycerol stearate and Ceteareth-25 (C16-18 alcohols, ethoxylated 25 mol EO average molar ratio) to the formulation results in higher values of hardness and lower values of adhesive strength, which consequently has less impact on the spreadability of the product [70]. In the studied macroemulsions, the same type of emulsifier and its concentration (4% wt.) was used. Formulations differed in the type and content of the oil phase. The serum (S), in which the oil phase was 25% (liquid lipid 20% wt., semisolid lipids 5% wt.), had the lowest value of hardness and the highest adhesive strength. In the case of the balm (oil phase proportion: liquid lipid 10% wt., semisolid lipids 10% wt.) and cream (proportion of oil phase: liquid lipid 15% wt., semisolid lipids 7% wt. and solid lipids 4% wt.), which contain higher concentrations of the semisolid and/or solid lipids, higher values of hardness and lower values of adhesion force were observed. Moreover, the addition of nanoemulgel (NG) to all emulsion systems leads to a decrease in its hardness and an increase in adhesion strength. Those data are in accordance with rheological analysis results. Serum, as well as the hybrid systems based on it, had the lowest viscosity value. The addition of nanoemulgel, the system with the lowest viscosity values (1.20 ± 0.01 Pa·s), into the hybrid systems decreased the viscosity of the formulations. On the other hand, bigel was characterized by the highest value of viscosity (3.28 ± 0.17 Pa·s), hardness (233.8 g), and the lowest adhesion force (−66.2 g). Texture measurements have shown that the tested formulations are different in terms of textural properties, and the hardness and strength of adhesion of emulsion formulations and hybrid combinations depend on the content and composition of the oil phase in the formulation and the content of nanoemulgel.

### 4.2. Cytotoxicity Test of Formulations

Keeping in mind the often-reported side effects of available *psoriasis* treatments and the discomfort of patients, it is important to highlight that prepared formulations were well tolerated. This was confirmed in in vitro assays performed on the immortalized human keratinocyte cell line HaCaT. To obtain safe formulations, their composition has been optimized to reduce cytotoxicity. Among the applied emulsifiers, several whose cytotoxicity did not exceed 50% were identified, even for the highest tested concentration. Based on cell viability assay results, the following emulsifiers were selected for further studies: cetearyl glucoside (and) cetearyl alcohol; olive oil polyglyceryl-6 esters (and) sodium stearoyl lactylate (and) cetearyl alcohol; cetearyl olivate (and) sorbitan olivate. As the hyaluronic matrix showed much lower cytotoxicity than the carbomer matrix, it was also chosen as the safe component of the obtained formulations. Even more significantly, the toxicity of ursolic acid was reduced in the prepared formulations. Only in the case of the bigel and serum was a different tendency observed. These two systems exhibited significantly larger IC25% and IC50% values. This decreased cytotoxicity might be related to the increased retention of UA in these formulations compared to direct application to the medium and may have the potential to translate to longer persistence at the site of application. On the other hand, the enhanced cytotoxicity of the bigel and serum may be related to the increased release and/or bioavailability of ursolic acid from these preparations. Nonetheless, regardless of the cytotoxicity determined in the MTT assay, the essential factor in choosing the best composition is the magnitude of a desired biological effect within the range of well-tolerated formulation concentrations, not the cytotoxicity at high concentrations itself. Therefore, the safety and biological activity of the optimized formulations are the focus of our ongoing research.

### 4.3. Release of Ursolic Acid from Formulation and Kinetic Analysis

The results of ursolic acid release from the elaborated formulations did not show an initial burst followed by a slow drug release. Among the topical formulations studied, the rate was the fastest for the O/W emulsion and the slowest for the bigel system. These results may depend on both the physicochemical properties of the vehicles and of the drug. The viscosity of vehicles could be one of the factors affecting active substance release since it may reduce the diffusion rate of the drug from the formulation. The drug can diffuse from the inner part of the carrier to the external environment while being delayed by the carrier’s material resistance and external viscosity [71,72]. It was also found that the rate of release generally increases from smaller particles since a small-particle system has a large total surface area where drug diffusion can occur [73,74]. Another parameter influencing drug release from the vehicle can be the lipophilic nature of a substance, which strongly affects its affinity to vehicle formulation. The partition coefficient of a drug may strongly define its affinity to its vehicle formulation. Ursolic acid is a strongly hydrophobic substance, as seen from the logP value = 6.58 [75]. In general, while the content of lipophilic ingredients in the vehicle increases, the release of a lipophilic drug decreases compared to a hydrophilic drug [36,71,73,74,76].

Additionally, the long release duration of the drug can be attributed to high initial loading concentrations of the drug as well as the interaction of the drug with the gelator (hydrogelators and oleogelators). The drug can interact or absorb with the gelators, which may have a role in extending the duration of drug release [77]. Moreover, there can be steric hindrance on the drugs tethered by the hydrogel matrix [78]. The slow drug release may be caused by the physical barrier from the vehicle material. In our studies, slower drug release occurred with higher gelator concentrations in the formulation (macroemulsion < hybrid, nanoemulgel < bigel). This phenomenon seems to be reasonable if we consider the fact that bigels are formulations that constitute two-phase structured systems obtained by mixing hydrogel and oleogel [79].

It also should be emphasized that bigels are characterized by the highest viscosity and the highest content of the oil phase, which results in the greatest affinity of the drug to the substrate. Because in the case of other analyzed formulations, such dependence was not observed, one of the possible explanations could be provided if we consider that the bulk delivery conditions could affect the drug release. Some of these features that influence release are listed as follows: polymer degradation, polymer or drug dissolution in the external medium, possible degradation of excipients or stabilizers, creation of hydrostatic pressure inside the system, coalescence of drug carriers, variation in pH or temperature induced by nonprogrammed stimuli, or environmental phenomena [72,80,81,82,83,84,85,86,87,88].

Based on the value of the coefficient of determination R^2^ (Table 8), it could be concluded that Higuchi’s model described the kinetics of ursolic acid release from the NG-S hybrid combination with a very suitable approximation. According to this model, the diffusion process is the factor controlling ursolic acid release from the carriers. This equation, based on principles of diffusion as expressed by Fick’s first law of diffusion, describes the release of the active substance from topical dosage forms such as gels, creams, and ointments. This model suggests a pure diffusion release mechanism of the active substance from a vehicle, with no erosion or swelling of the matrix [89]. For the remaining formulations, the most suitable model is Korsmeyer–Peppas. This model suits the best when approximating the kinetics of UA release from polymer matrices such as the nanoemulgel and bigel. This model considers the occurrence of processes such as water diffusion, swelling, gel formation, active substance diffusion, and matrix dissolution. Moreover, the Korsmeyer–Peppas model can be used as a decision parameter between the Higuchi and zero-order models [90]. However, in the case of release kinetics, not only the physicochemical form of the formulation but also the chemical nature of the drug and its affinity for the carrier is important [36,71,73,74,76]. The exponent n in the Korsmeyer–Peppas model in the case of macroemulsion and nanoemulgel was in the range of 0.5 < n < 1.0, which indicated that the mechanism characterizing the release of the triterpene acid was anomalous diffusion, different from Fick diffusion (indicates non-Fickian release), i.e., a combination diffusion and influence of the acceptor fluid on the carrier (e.g., erosion, swelling). On the other hand, for large particles, the value of n is <0.5, so the main role in the kinetics of ursolic acid release is Fick diffusion [35,91].

According to the literature reports, the release of ursolic acid occurred in accordance with the Krosmeyer–Peppas model in the case of nanoemulsions as carriers [92] and Higuchi in the case of dendrimeric nanoparticles [93].

## 5. Conclusions

Various topical ursolic acid-loaded formulations, such as nanoemulgel, macroemulsions (serum, cream, balm), oleogel, and hybrid systems, such as nanoemulgel–macroemulsion and nanoemulgel–oleogel (bigel), have been prepared as products for *psoriasis* treatment. Based on the results of in vitro cytotoxicity studies of the individual raw materials, the best composition of the formulation was selected. Moreover, the safety of the elaborated formulations was confirmed in in vitro assays performed on the immortalized human keratinocyte cell line HaCaT. All of the studied UA-loaded systems were well tolerated by keratinocyte cells and had suitable pH values, stability over time, and durability of ursolic acid in formulations. The obtained formulations, thanks to the combination of physicochemical properties of both forms, such as macroemulsion and nanoemulgel, were characterized by apparent viscosity, ensuring the appropriate time of contact with the skin, ease of spreading, pleasant sensory sensations, soft consistency, and quick absorption. These properties may encourage people suffering from *psoriasis* to use it regularly and frequently and consequently increase the therapeutic effect. They can also facilitate the transportation of the active substances, especially since the release of ursolic acid from each of the formulations did not show an initial burst, followed by a slow controlled release according to the Korsmeyer–Peppas and Higuchi models. As a result, due to such properties, the elaborated systems could be considered suitable vehicles to administer triterpene to psoriatic skin.

## 6. Patents

K. Nowak, A. Kulawik-Pióro, E. Lasoń, M. Malinowska, M. Miastkowska, E. Sikora, K. Śliwa, A method of producing a nanogel composition with care, cosmetic and therapeutic properties and a nanogel composition with care, cosmetic and healing properties, P.441627, 2022.

## Figures and Tables

**Table 1:** Components used in the studies.

Component Groups	Commercial Name	INCI Name	Suppliers
Emulsifiers	Crodesta SL 40	Aqua (and) sucrose cocoate and alcohol	Croda Cracow Poland
	Emulgade PL 68/50	Cetearyl glucoside (and) cetearyl alcohol	BASF Warsaw Poland
	Olivatis 18	Olive oil polyglyceryl-6 esters, sodium stearoyl lactylate, cetearyl alcohol	Alfa Sagittarius Cracow Poland
	Olivem 1000	Cetearyl olivate, sorbitan olivate.	Hallstar HSH Chemie Sp.z o.o Warsaw Poland
	Emulgin SML 20	Polysorbate 20	BASF Warsaw Poland
	Plantacer 2000 UP	Decyl glucoside	BASF Warsaw Poland
Hydrogelators	Carbopol ETD 2050	Carbomer	Lubrizol Warsaw Poland
	Sodium hyaluroniate 1.5–2.0 MDa high molecular weight	Sodium hyaluroniate	Alfa Saggittarius Cracow Poland
Oleogelators	Span 60	Sorbitan stearate	Croda Cracow Poland
	Span 80	Sorbitan oleate	Croda Cracow Poland
	Tween 80	Polysorbate 80	Croda Cracow Poland
	Aerosil 200	Silica	Evonik Warsaw Poland
	Aerosil R 816	Silica cetyl silylate	Evonik Warsaw Poland
Plant extracts	Aloe vera juice	*Aloe vera barbadensis* leaf juice	Provital Cracow Poland
	Horse chestnut seed extract	*Aesculus hippocastanum* (Horse chestnut) seed extract	
	Prunus Persica fruit extract	Glycerin (and) water (and) Prunus Persica (peach) fruit extract (and) sodium benzoate (and) potassium sorbate	
	Chia seed extract	*Salvia hispanica* seed extract	
Liquid lipids	Raspberry seed oil	*Rubus idaeus* (raspberry) seed oil	Ol’Vita Marcinowice Poland
	Borage seed oil	*Borago officinalis* (borage) seed oil	
	Tamanu seed oil	*Calophyllum inophyllum* (tamanu) seed oil	
	Sweet almond oil	*Prunus amygdalus dulcis* (sweet almond) oil	
	Safflower oil	*Carthamus tinctorius* (safflower) oil	
	Rice bran oil	*Oryza sativa* (rice) bran oil	
	Coconut oil	*Cocos nucifera* (coconut) oil	
	*Cannabis sativa seed oil*	*Cannabis sativa seed oil*	
	Carrot seed oil	*Daucus carota sativa* (carrot) seed oil	
	Sunflower oil	*Helianthus annus* (sunflower) seed oil	
	Myritol 318	Caprylic/capric triglyceride	Croda Cracow Poland
Semisolid lipids	Shea butter	Butyrospermum Parkii butter	Croda Cracow Poland
	Cocoa butter	*Theobroma cacao* (cocoa) seed butter	Croda Cracow Poland
	Murumuru butter	*Astrocaryum murumuru* seed butter	Alfa Sagittarius Cracow Poland
Solid lipids	Cutina CBS	Glyceryl stearate (and) cetearyl alcohol (and) cetyl palmitate (and) cocoglycerides	BASF Warsaw Poland
Preservatives	Dermosoft 1388	Aqua (and) glycerin (and) sodium levulinate (and) sodium anisate	Evonik Warsaw Poland
	Geogard Ultra^®^	Gluconolactone, sodium benzoate	Arxada Basel, Switzerland
Active ingredient	Ursolic acid	Ursolic acid	Merck Warsaw Poland

**Table 2:** General composition of ursolic acid-loaded nanoemulgel.

Phase	Component	Concentration (% wt.)
A	Aqua	30.0
	Hydrogelator	1.0
B	Emollient	10.0
	Emulsifier	4.0
	Ursolic acid	0.01–0.5
C	Preservative	2.5
	Aqua	up to 100

**Table 3:** General composition of macroemulsion formulations.

Phase	Component	Serum	Face Cream	Body Balm
		Concentration (% wt.)		
A	Aqua	Up to 100	Up to 100	Up to 100
	Plant extracts	10.0	15.0	5.0
	Preservative	2.5	2.5	2.5
B	Liquid lipid (vegetable oils, medium-chain triglycerides)	20.0	15.0	10.0
	Semisolid lipids (vegetable butters)	5.0	7.0	10.0
	Solid lipids (natural waxes, fatty alcohols)	−	4.0	−
	Emulsifier	4.0	4.0	4.0
	Ursolic acid	0.01–0.5	0.01–0.5	0.01–0.5

**Table 4:** General composition of oleogel.

Phase	Component	Concentration (% wt.)
A	Oleogelator 1	6.0
	Oleogelator 2	6.0
	Liquid lipids	88.0
	Ursolic acid	0.01–0.5

**Table 5:** General composition of hybrid systems.

Nanoemulgel (NG)	Formulation (F)			
	Serum (S)	Cream (C)	Balm (B)	Oleogel (O)
Ratios of NG:F (*w*/*w*)	NG:S	NG:C	NG:B	BG
5:95	−	−	−	BG-1
10:90	NG:S-1	NG:C-1	NG:B-1	BG-2
20:80	NG:S-2	NG:C-2	NG:B-2	BG-3
30:70	NG:S-3	NG:C-3	NG:B-3	−
40:60	NG:S-4	NG:C-4	NG:B-4	−
50:50	NG:S-5	NG:C-5	NG:B-5	−
60:40	NG:S-6	NG:C-6	NG:B-6	−
70:30	NG:S-7	NG:C-7	NG:B-7	−
80:20	NG:S-8	NG:C-8	NG:B-8	−
90:10	NG:S-9	NG:C-9	NG:B-9	−

**Table 6:** The average particle size, viscosity, and pH of the fresh formulations (t = 0) and after 3 months.

Sample	pH		Average Particle Size (μm)		Viscosity (Pa∙s) for γ = 50 s^−1^, T = 25 °C	
	t = 0	t = 3 Months	t = 0	t = 3 Months	t = 0	t = 3 Months
NG	5.00 ± 0.0	5.05 ± 0.0	101·10^−3^ ± 0.002	110·10^−3^ ± 0.003	1.20 ± 0.01	1.25 ± 0.01
S	6.00 ± 0.0	6.00 ± 0.0	1.25 ± 0.04	1.15 ± 0.03	2.50 ± 0.17	2.55 ± 0.15
NG:S-4	5.09 ± 0.1	5.20 ± 0.0	1.25 ± 0.05	1.10 ± 0.06	2.26 ± 0.09	2.30 ± 0.10
NG:S-5	5.00 ± 0.1	5.10 ± 0.0	1.26 ± 0.08	1.23 ± 0.03	2.15 ± 0.08	2.20 ± 0.06
NG:S-6	5.40 ± 0.0	5.50 ± 0.0	1.39 ± 0.03	1.21 ± 0.08	2.00 ± 0.06	2.10 ± 0.04
C	6.00 ± 0.1	6.05 ± 0.0	2.61 ± 0.10	3.34 ± 0.18	2.98 ± 0.09	2.72 ± 0.10
NG:C-6	5.00 ± 0.0	5.25 ± 0.0	2.71 ± 0.16	3.06 ± 0.12	2.45 ± 0.09	2.50 ± 0.10
NG:C-7	5.00 ± 0.1	5.10 ± 0.0	2.68 ± 0.08	3.12 ± 0.09	2.39 ± 0.04	2.40 ± 0.03
B	6.05 ± 0.1	6.11 ± 0.1	2.23 ± 0.11	2.86 ± 0.10	2.90 ± 0.08	2.80 ± 0.11
NG:B-4	4.76 ± 0.1	4.88 ± 0.1	2.16 ± 0.05	2.95 ± 0.03	2.60 ± 0.10	2.70 ± 0.12
NG:B-5	4.94 ± 0.0	4.99 ± 0.0	1.70 ± 0.09	1.03 ± 0.04	2.34 ± 0.07	2.40 ± 0.15
O	4.05 ± 0.0	4.10 ± 0.0	−	−	3.36 ± 0.06	3.47 ± 0.08
BG-1	4.69 ± 0.1	4.73 ± 0.1	34.35 ± 1.73	35.75 ± 2.15	3.28 ± 0.17	3.97 ± 0.16

**Table 7:** Cytotoxicity of ursolic acid and full cosmetic formulations determined with MTT assay on HaCaT cells. IC25%—percentage concentration of formulation that causes a decrease in cell viability by 25%. IC50%—percentage concentration of formulation that causes a decrease in cell viability by 50%. The concentration of ursolic acid present at the given concentration of the formulation is marked in parentheses.

Sample	IC50% (UA Concentration µM)	IC25% (UA Concentration µM)
Ursolic acid	-(>50 µM)	-(>25 µM)
NG	>9.13% (>200 µM)	>9.13% (>200 µM)
BG	>0.91% (>20 µM)	>0.05% (>1 µM)
B	>18.27% (>400 µM)	>2.28% (>50 µM)
S	>1.14% (>25 µM)	>0.23% (>5 µM)
C	>18.27% (>400 µM)	>9.13% (>200 µM)
NG:S-5	>9.13% (>200 µM)	>2.28% (>50 µM)
NG:B-4	>18.27% (>400 µM)	>9.13% (>200 µM)
NG:C-7	>18.27% (>400 µM)	>18.27% (>400 µM)

**Table 8:** The kinetic model parameters corresponding to the release results.

Model	Parameter	Formulation			
		S	NG	NG:S	BG
Zero order $C_{t}=K_{0}\cdot t$	R^2^	0.9743	0.9844	0.9569	0.9741
	K_0_ (mg/h)	0.1089	0.0793	0.0803	0.0582
First order $log{(100-C}_{t})=\frac{-K_{1}\cdot t}{2.303}$	R^2^	0.9851	0.9883	0.9674	0.9804
	K_1_ (h^−1^)	0.04398	0.03063	0.0311	0.02189
Higuchi $C_{t}=K_{H}\cdot \sqrt{t}$	R^2^	0.9867	0.9754	0.9919	0.9878
	K_H_ (mg/h^1/2^)	0.3481	0.2506	0.2597	0.1859
Korsmeyer–Peppas ${logC}_{t}={logK}_{KP}+n\cdot logt$	R^2^	0.9884	0.9899	0.9823	0.9912
	K_HP_ (h^−n^)	8.5349	7.2443	8.3772	6.9374
	n	0.6119	0.5313	0.4932	0.4485

## Data Availability

The authors confirm that the data supporting the findings of this study are available within the article.

## Abbreviations

B	balm
C	cream
DMEM	Dulbecco’s modified Eagle’s medium
DLS	dynamic light scattering
FBS	fetal bovine serum
ME	macroemulsion
NE	nanoemulsion
NG	nanoemulgel
O	oleogel
PBS	phosphate-buffered saline
PDI	polydispersity index
S	serum
SEM	scanning electron microscope
TPA	profile texture analysis
TSI	Turbiscan stability index
UA	ursolic acid
